# Full Mouth Reconstruction of a Bruxer with Severely Worn Dentition: A Clinical Report

**DOI:** 10.1155/2015/531618

**Published:** 2015-07-01

**Authors:** Somayeh Zeighami, Hakimeh Siadat, Sakineh Nikzad

**Affiliations:** ^1^Dental Research Center and Department of Prosthodontics, School of Dentistry, Tehran University of Medical Sciences, Tehran, Iran; ^2^Dental Implant Research Center and Department of Prosthodontics, School of Dentistry, Tehran University of Medical Sciences, Tehran, Iran

## Abstract

Tooth wear is attributed to several factors many of which often remain unidentified. Management of tooth wear is challenging in preventive and restorative dentistry. Correct assessment of occlusal vertical dimension, interocclusal rest space, and centric relation records are critical for successful treatment. In order to evaluate different treatment modalities and select the treatment of choice some information can be obtained from study casts and diagnostic wax-up. In order to achieve a predictable and desirable result, a systematic approach may be helpful. This paper describes the full mouth rehabilitation of a 36-year-old bruxer with severely worn dentition and other dental problems such as unfavorable restorations. A diagnostic work-up was performed and provisional restorations were made; then, they were clinically evaluated and adjusted based on the criteria dictating esthetics, phonetics, and vertical dimension. After endodontic therapy, clinical crown lengthening was performed. Two short implants were inserted in the posterior mandible. Custom-cast dowel cores and metal-ceramic restorations were fabricated and a full occlusal splint was used to protect the restorations. We ensured stable contacts on all teeth with equal intensity in centric relation and anterior guidance in accord with functional jaw movements.

## 1. Introduction

According to evidence tooth wear is growing up from aspects of prevalence and severity both in seniors who are living longer and adults. Recognition of tooth wear's presence and determination of the activity status of the process are essential to programing management strategies and treatment processes [1]. Attrition is defined as a loss of tooth structure caused by tooth-to-tooth friction without any intervening substance. Occlusal and incisal attrition may occur during deglutition (physiological wear) and may be severe if parafunctional activities such as bruxism and clenching habits exist (pathologic wear). Shiny dental surface and well-defined facets are considered reliable signs of attrition that usually match facets of teeth in the opposing arch in eccentric occlusion, especially in anterior antagonists [2–4]. Such facets are mostly seen on functional surfaces (occlusal and incisal) of teeth but may affect buccal and palatal surfaces of the teeth in the anterior mandible and maxilla when a deep vertical overlap is present [5]. In attrition the wear rate in the upper and lower jaw are equal because intimate contact of opposing surfaces can result in matching wear facets [6]. Tooth wear is attributed to several factors many of which often remain unidentified [7]. Pulpal pathologies, impaired occlusal function, and esthetic problems may result from excessive occlusal attrition [8].

Management of tooth wear and attrition is an interesting subject in preventive and restorative dentistry [9]. After definitive diagnosis, full mouth reconstruction concomitant with controlling causative factors will be one of the treatment options [3]. The occlusion vertical dimension (OVD), the interocclusal rest space (IRS), and centric relation (CR) records are critical for successful treatment. In order to achieve a predictable and desirable prognosis, a systematic approach may be helpful [10]. Tooth eruption and alveolar bone growth can compensate for the loss of OVD in some cases. Following tooth wear, an adaptive process continues in the alveolar bone to compensate for the loss of tooth structure and restore the OVD. Therefore, OVD is a sensitive subject and should not be manipulated without a careful assessment [11, 12].

Management of dentition attrition is challenging and difficult to accomplish. Evaluation of vertical dimension plays a critical role in comprehensive treatment planning and requires mounted study casts and diagnostic wax-up. Clinical assessment of patient after cementation of provisional fixed restorations or wearing a diagnostic splint/interim removable prosthesis can help in determination of the OVD [13]. This study discusses the phases of prosthodontic rehabilitation, from diagnosis to final treatment, of a 36-year-old bruxer with severely worn dentition.

## 2. Case Report

### 2.1. Chief Complaint

A 36-year-old woman referred to the Department of Prosthodontics of the Faculty of Dentistry of Tehran University of Medical Sciences, Tehran, Iran, for prosthodontics treatment of her worn teeth. The patient's chief complaint was chewing deficiency and speech problems.

### 2.2. History

The patient had a history of low-level estrogen controlled by 5 mg Prednisolone daily for the past 20 years. No other remarkable findings were found in her medical history. The patient had a history of endodontic, restorative, and prosthodontic treatment. She first noticed tooth wear 2 years ago.

### 2.3. Examination

**Figure 1:**
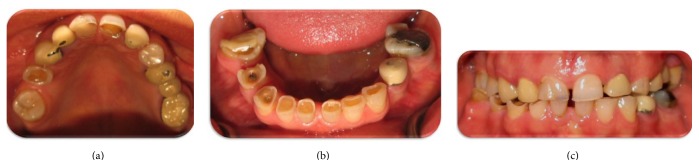
Pretreatment intraoral view.

The patient had no asymmetry and had competent lips and no signs or symptoms (pain, limited range of jaw opening, or clicking) of temporomandibular joint disorder (TMD) were detected. Initial evaluation of the patient revealed parafunctional habits of bruxism and clenching. Clinical examinations revealed severe attrition of the anterior and posterior teeth. Well-defined facets matching those on the opposing teeth in eccentric occlusion were also detected. The severe tooth wear was attributed to parafunctional habits, unsuitable restorations, and posterior dental interferences. A discrepancy between the centric occlusion (CO) and maximum intercuspal position (MIP) was found when she was guided into CR using the bimanual technique. The most prominent interference upon sliding the mandible from CO to the anterior position was tooth #30. There was no anterior guidance for posterior eccentric movements. The patient had an acceptable oral hygiene and there were no periodontal problems (Figure 1).

**Figure 2:**
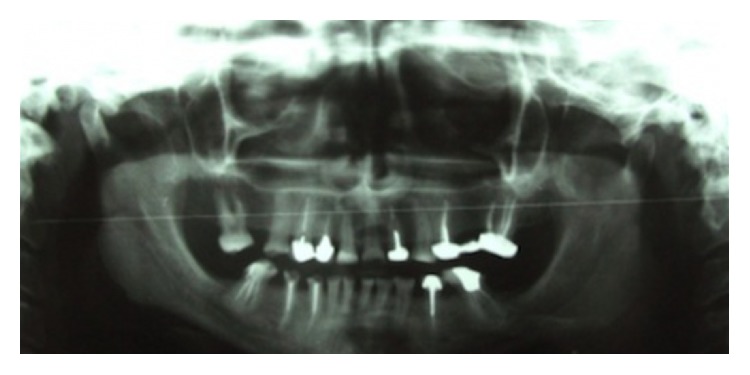
Panoramic radiographic image before treatment.

In patient's panoramic radiography it was found that the second premolars and the second and third molars were missing in all 4 quadrants and teeth #6, 7, 10, 12, 14, 19, 21, 27, 28, and 30 had unacceptable root canal therapy (RCT) (Figure 2).

### 2.4. Diagnostic Procedures

After the vertical dimension was clinically assessed, physiologic rest position was determined by facial measurements between nose tip and chin and confirmed by phonetics [14]. The interocclusal distance was found to be approximately 4 mm, and the OVD could be restored by approximately increasing it by 1 mm. In addition, the tooth wear resulted in protrusive deviation of the mandible. By guiding the mandible into centric relation, there was space in the anterior region for rehabilitation [6]. By grinding tooth #30, occlusal interferences were removed and CO and MIP were then equal.

Oral hygiene was instructed. Prior to definitive treatment, diagnostic plaster casts (Moldano Dental Stone, Bayer Co., Leverkusen, Germany) were obtained from alginate impressions (Tropicalgin, Zhermack, Badia Polesine, Rovigo, Italy). The bite registration procedure was accomplished using an acrylic anterior deprogramming device (Pattern Resin LS, GC America, ALSIP, IL, USA) in the anterior region and baseplate wax (Cavex Setup Regular Modelling Wax, Cavex Holland BV, Haarlem, Netherlands). The mandible was guided into CR by bimanual manipulation technique. To confirm the record, a small amount of zinc oxide eugenol paste (Luralite, Kerr Corp., Orange, CA, USA) was placed on the wax over each indented area, and the mandible was held in CR until the paste had set. This record and an arbitrary facebow (Dentatus Facebow; Dentatus AB, Spanga, Sweden) were used to mount the casts on a semiadjustable articulator (Dentatus ARH-Type; Dentatus AB, Spanga, Sweden). The condylar guidance on the articulator was set at average. The incisal pin was adjusted for a 1 mm opening. The curves of Spee and Wilson as well as the orientation of the occlusal plane were determined using a Broadrick occlusal plane analyzer. After a diagnostic wax-up at this new OVD, a new cast was made (with duplication of diagnostic wax-up) and via autopolymerizing acrylic resin (Tempron, GC Europe) provisional crowns were fabricated using a vacuum formed matrix (Drufolen H; Dreve Dentamid GmbH, Unna, Germany). Teeth were prepared using a putty index made from the diagnostic wax-up. During preparation the tooth pulp of teeth #3 and #22 was exposed and revealed that the clinical crown lengths of teeth #5, 8, 9, 11, 23, 24, 25, and 26 were not sufficient for fixed restorations. The provisional fixed restorations were cemented using temporary cement (Temp Bond, Kerr Corp., Orange, CA, USA). The patient used these provisional restorations for 2 months to check the proposed vertical dimension. For two months, provisional restorations were adjusted and used as a guide for definitive oral rehabilitation. During this period, the patient's condition and functions such as muscle tenderness, discomfort of TMJ, mastication, range of the mandibular movements, swallowing, and speech were evaluated.

A treatment plan was developed with the aim of improving occlusion, restoring masticatory function, and improving the patient's appearance. During the following visit, treatment options were discussed with the patient, including root canal therapy or retreatment, periodontal therapy (including crown lengthening in all regions), and prosthetic treatment (metal ceramic restorations (MCRs)).

### 2.5. Endodontic and Periodontal Procedures

The first phase of treatment was RCT of exposed teeth and teeth with insufficient crown lengths and retreatment (ReRCT) of teeth with unacceptable RCTs. During ReRCT of teeth #19 and 30 it became apparent that these teeth had vertical fractures and had to be extracted. These teeth were extracted and implant placement was discussed; the patient consented to implant placement of teeth #19, 20, 29, and 30.

After completion of RCT, crown-lengthening surgery in all regions was performed using a vacuum shell guide according to the diagnostic wax-up. After 1 week, the provisional restorations were adjusted according to the new margins.

### 2.6. Implant Placement Phase

According to the diagnostic wax-up in region of teeth #19, 20, 29, and 30 a radiographic stent was fabricated, coated with barium sulfate (Barium Sulfate, Daroupakhsh Co., Tehran, Iran), and drill holes were filled with gutta-percha. Cone-beam CT scan (CBCT) (QR SRL Company, Verona, Italy) was used for evaluation of dental implant positions. CBCT revealed 4 mm bone height in the first molar regions and thus implant placement was not possible in these regions but in the premolar regions bone height was 10 mm and short implant with 8 mm length could be placed. After determining the position of implants, implant placement surgery was scheduled. A new stent was fabricated for surgery and two months after teeth extraction, two implants were placed in the right and left mandibular segments to replace the mandibular right and left first molars (ITI Implants, Regular CrossFit Connection 4.1 mm diameter, 8 mm long, Straumann AG, Waldenburg, Switzerland). After 4 months, the dental implants were ready for loading.

### 2.7. Prosthodontic Treatment

During the period of implant osseointegration, according to the wax-up index and ideal occlusal plane, post and core patterns for all teeth that required build-up after root canal therapy were prepared; custom-cast dowel cores (VeraBond, AlbaDent Co., USA) were fabricated and cemented with Panavia F2 (Kuraray Noritake Dental Inc.). Tooth preparation with a circumferential sloping shoulder margin configuration was performed on all teeth. After completion of the preparations, alginate impressions (Tropicalgin, Zhermack, Badia Polesine, Rovigo, Italy) for provisional restorations were made and new laboratory-processed provisional restorations (ALIKE, GC America, ALSIP, IL, USA) were fabricated and cemented with zinc oxide temporary cement (Temp Bond; Kerr Corp., Orange, CA, USA). The canine protected occlusion was established bilaterally.

**Figure 3:**
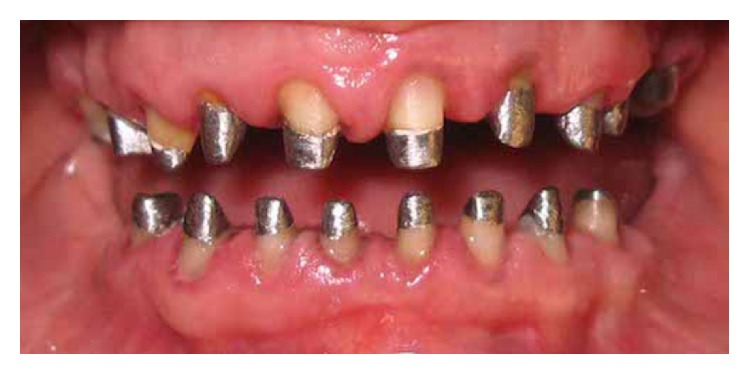
Prepared teeth before impression making.

After 4 months a screw type provisional restoration using temporary abutments for each implant was made. After adjusting provisional restorations, hydrocolloid impressions were made from provisional restorations and casts were transferred to the Denar Mark II articulator (Whip Mix Corporation, Louisville KY, USA) using the Denar Slidematic facebow (Whip Mix Corporation, Louisville KY, USA) and CR record. Adjusted occlusion was transferred to customized anterior guide table, which was made with acrylic resin (Pattern Resin LS, GC America, ALSIP, IL, USA). Before this procedure, condylar guidance was adjusted by lateral interocclusal records made with wax wafers (Cavex Setup Regular Modelling Wax, Cavex Holland BV, Haarlem, Netherlands). After completion of teeth preparations (Figure 3), the final impressions were made with 2-step impression technique (Putty and light body impression materials) (Speedex, Coltene AG, Altstatten, Switzerland) in a stock tray.

**Figure 4:**
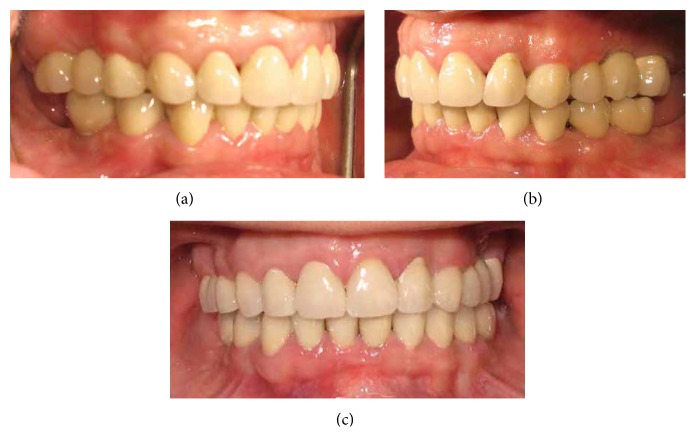
Views of MCRs in the mouth.

Fixture level implant impressions were made simultaneously with teeth using the open tray technique. The casts were mounted (cross mount technique) on the articulator using interocclusal registrations recording CR by guiding the mandible via bimanual manipulation and anterior deprogramming device with baseplate wax in the posterior area. According to the crown height space and fixture angulation abutment selection was performed (Regular CrossFit Connection, Cementable Abutment, diameter 5.0 mm, gingiva height 1.0 mm, abutment height 4.0 mm ITI Implants, Straumann AG, Waldenburg, Switzerland). A full contour wax-up was accomplished for the MCRs. Then cut-back was performed according to the index; next, the metal frameworks (VeraBond 2, AlbaDent Co., USA) were fabricated. The frameworks were evaluated radiographically and intraorally for fit, retention, and stability. Porcelain (Dentsply Ceramco, Burlington, NJ) was baked to complete the crowns. The lingual contours of the maxillary incisors were adjusted according to the anterior guide table. Finally, the occlusion of restorations was adjusted so that equal-intensity centric contacts were established on all teeth and canine guidance precluded all posterior teeth in eccentric jaw movements. Because the patient's anterior guidance table was used in the production of definite restoration, the amount of occlusal adjustment on the lingual surface of maxillary anterior teeth was minimal. The canine protected the posterior teeth from excursive force and wear, and posterior teeth supported the bite force. The MCRS were provisionally cemented using temporary cement (Temp Bond; Kerr Corp., Orange, CA, USA) (Figure 4).

**Figure 5:**
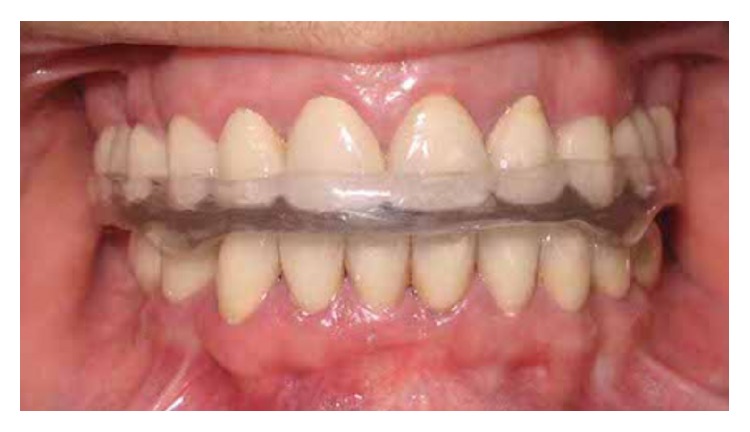
Frontal view of occlusal splint in the mouth.

**Figure 6:**
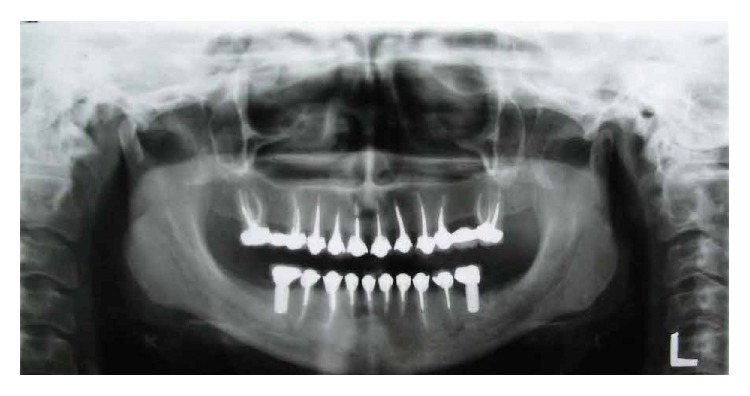
Posttreatment panoramic radiography.

Oral hygiene instructions were given. A hard acrylic resin full occlusal splint (Acropars, Marlic Co., Tehran, Iran) was fabricated for night use to prevent parafunctional occlusal wear. The occlusal splint was relieved in implant regions (Figure 5). Minor adjustments were required at first postinsertion visits. Posttreatment panoramic radiography of the patient is shown in Figure 6.

**Figure 7:**
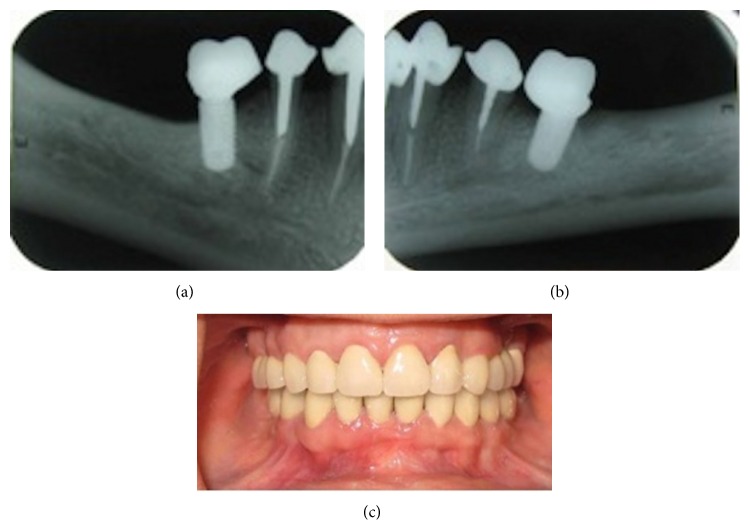
Intraoral view and periapical radiographies of implants after one-year follow-up.

After 2 months, the temporary cement was changed to zinc phosphate cement (Richter and Hoffmann's, Berlin, Germany), and the patient was placed on a 6-month recall. The 1-year evaluation of the esthetics and function of the restorations showed no evidence of temporomandibular joint problems, fractures in the teeth, or MCRs. Implant evaluations did not show peri-implant mucositis or unusual bone loss. Intraoral view and periapical radiographies of implants after one-year follow-up are revealed in Figure 7. No problem was found in clinical and radiographic examinations after one-year follow-up.

## 3. Discussion

Tooth surfaces can undergo erosion, attrition, and abrasion [15]. Tooth wear may be generalized or localized to incisors and canines [16]. Tooth attrition has a multifactorial etiology. The number of clinical controlled trials on restorative and prosthodontic approaches in this respect is sparse. Insufficient evidence regarding the long-term clinical outcome of treatment can complicate clinical decision making [17]. The etiology of wear should be determined before any intervention [16]. Niswonger, cited by Tallgren, found that 80% of patients with severe tooth wear have a normal interocclusal rest space. The distribution of wear in the dentition is usually uneven, evidenced by the difference between the anterior and posterior teeth [18]. Posterior occlusal prematurity may cause increased function of anterior teeth, resulting in increased attrition [8].

The trial period of fixed provisional prostheses is 2–6 months [8, 12, 19–22]. In this case provisional fixed restorations were evaluated for 2 months. Clinical judgment plays a major role in the assessment of OVD in rehabilitation. Phonetics, interocclusal distance, swallowing, and patient preferences can be used for measurements to correct OVD [22, 23].

Four options are available for treatment of severely worn teeth.

(1) Pin-retained full-gold restorations: the exposed dentin can be restored by using parallel pin retention with no significant increase in OVD. However, it may not be esthetically acceptable in the anterior region.

(2) Increasing the OVD: increased OVD may improve esthetics. However, excessive stress may result.

(3) Crown-lengthening procedures: surgical exposure of adequate tooth structure may be required for retention and esthetic contouring.

(4) Pulp extirpation, endodontics, and coping construction [11]. 

In present case three last methods were used to provide the space needed for restorations.

In our case the cone-beam CT was used to evaluate the meticulous placement of the implants. Traditional radiographs provide only a 2D image. CBCT provides information in all three dimensions. CBCT images are sufficiently accurate for preimplant assessments [24–27]. A surgical guide is usually fabricated based on the results of computer analysis of the available bone. Proximity and location of the remaining teeth relative to the implant site and vital structures to be avoided are also taken into account. This information is extremely helpful for clinicians in selecting an optimal location to place the implants and is critical for improving the chances for a successful outcome [27, 28].

The shortened dental arch (SDA) concept, first discussed internationally by the Dutch prosthodontist Professor Käyser in 1981, has proven to be worth serious consideration in treatment planning for partially edentulous patients. A study on SDA (shortened dental arches) revealed that presence of the anterior and premolar teeth could fulfill the required criteria for a functional dentition. Excellent long-term functional outcomes have been reported in absence of molar support [29]. The SDA treatment approach is advantageous and does not contradict current occlusion concepts [30]. In this case one premolar and one molar in each quadrant were restored.

In order to assess the effect of occlusal disharmony on nocturnal bruxism a night guard can be used. It is also helpful for stress relief during nocturnal parafunction. However, the treatment plan should be able to tolerate greater forces. Centric contacts in CR occlusion and disocclusion of the posterior teeth guided by the anterior teeth in excursions are strongly recommended for fabricating a night guard (for the maxilla or mandible). Unlike the natural teeth, extrusion of implants does not occur if the occlusal contacts are not present. In patients with a posterior quadrant of implants supporting a mandibular fixed partial denture opposing the maxillary teeth, the occlusal surfaces of the maxillary night guard are removed over the implant crowns so no occlusal contacts are present and no occlusal forces are transferred to the implants [31].

In a study by Tawil et al., the bruxer group experienced more severe complications. However, no statistically significant difference was found in prevalence of complications between different bruxer groups [32]. In severely worn teeth, equal-intensity centric occlusal contacts must be achieved on all teeth. Furthermore, an anterior guidance occlusion must be established in accordance with normal functional jaw movements with all the posterior teeth out of occlusion during the eccentric jaw movements. Establishment of this occlusal pattern is more important in bruxers. Finally what is the most important in long-term success is periodic monitoring of etiologies included bruxism and steepen anterior guidance that restricts envelope of function those will cause recurrent wear on anterior teeth or destroying of anterior restorations. Any sign of recurrent wear should be identified and controlled as soon as possible [11].

## 4. Conclusion

Full mouth reconstruction in wear patients is one of the most common treatment options. The mystery of excellent prognosis is keyed to an accurate anterior guidance that is in harmony with the envelope of function and has sufficiency in posterior disclusion.
